# Vitamin-D-Binding Protein Contributes to the Maintenance of α Cell Function and Glucagon Secretion

**DOI:** 10.1016/j.celrep.2020.107761

**Published:** 2020-06-16

**Authors:** Katrina Viloria, Daniela Nasteska, Linford J.B. Briant, Silke Heising, Dean P. Larner, Nicholas H.F. Fine, Fiona B. Ashford, Gabriela da Silva Xavier, Maria Jiménez Ramos, Annie Hasib, Federica Cuozzo, Jocelyn E. Manning Fox, Patrick E. MacDonald, Ildem Akerman, Gareth G. Lavery, Christine Flaxman, Noel G. Morgan, Sarah J. Richardson, Martin Hewison, David J. Hodson

**Affiliations:** 1 Institute of Metabolism and Systems Research (IMSR), University of Birmingham, Birmingham B15 2TT, UK; 2 Centre for Endocrinology, Diabetes and Metabolism, Birmingham Health Partners, Birmingham B15 2TT, UK; 3 Centre of Membrane Proteins and Receptors (COMPARE), University of Birmingham and University of Nottingham, Midlands, UK; 4 Oxford Centre for Diabetes, Endocrinology and Metabolism, Radcliffe Department of Medicine, University of Oxford, Oxford OX3 7LE, UK; 5 Department of Pharmacology and Alberta Diabetes Institute, University of Alberta, Edmonton, AB T6G 2E1, Canada; 6 Institute of Biomedical and Clinical Science, University of Exeter Medical School, Exeter EX2 5DW, UK

**Keywords:** α cell, glucagon, GC-globulin, vitamin D, vitamin-D-binding protein, type 1 diabetes

## Abstract

Vitamin-D-binding protein (DBP) or group-specific component of serum (GC-globulin) carries vitamin D metabolites from the circulation to target tissues. DBP is highly localized to the liver and pancreatic α cells. Although DBP serum levels, gene polymorphisms, and autoantigens have all been associated with diabetes risk, the underlying mechanisms remain unknown. Here, we show that DBP regulates α cell morphology, α cell function, and glucagon secretion. Deletion of DBP leads to smaller and hyperplastic α cells, altered Na^+^ channel conductance, impaired α cell activation by low glucose, and reduced rates of glucagon secretion both *in vivo* and *in vitro*. Mechanistically, this involves reversible changes in islet microfilament abundance and density, as well as changes in glucagon granule distribution. Defects are also seen in β cell and δ cell function. Immunostaining of human pancreata reveals generalized loss of DBP expression as a feature of late-onset and long-standing, but not early-onset, type 1 diabetes. Thus, DBP regulates α cell phenotype, with implications for diabetes pathogenesis.

## Highlights

• Vitamin-D-binding protein (DBP) is highly expressed in pancreatic α cells
• Glucagon secretion and insulin tolerance are altered in mice lacking DBP
• DBP-null α cells possess an abnormal actin cytoskeleton and are dysfunctional
• DBP levels are decreased in α cells of donors with late-onset type 1 diabetes

## Graphical Abstract

**Figure:**
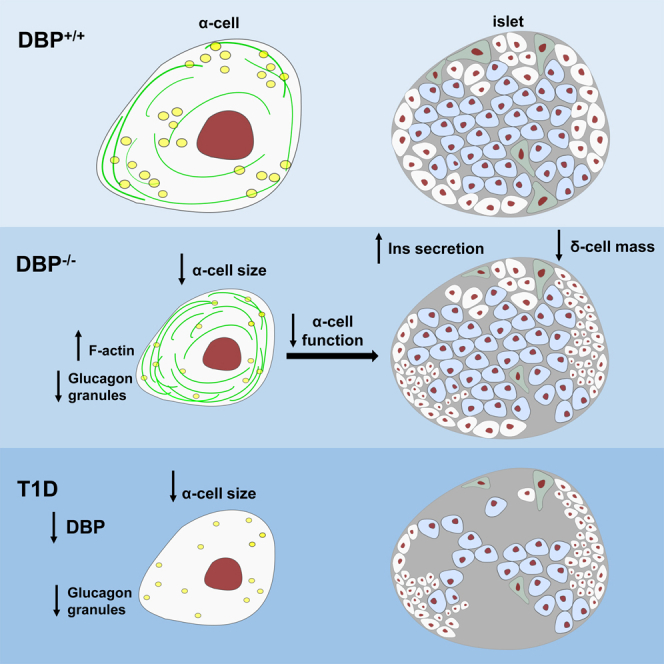

## Teaser

Viloria et al. show that vitamin-D-binding protein (DBP) contributes to insulin tolerance and circulating glucagon levels in mice. Loss of DBP leads to smaller, hyperplastic α cells, with increased abundance of F-actin microfilaments. These changes are associated with decreased α cell activity, re-distribution of glucagon granules, and impaired glucagon secretion.

## Introduction

Vitamin-D-binding protein (DBP), a 52- to 59-kDa protein also known as group-specific component of serum (GC-globulin), is the primary plasma carrier for circulating vitamin D and its metabolites (White and Cooke, 2000). *GC/Gc*, which encodes DBP, is present in the liver of all mammals (Feldman et al., 2017), in keeping with the function of this organ to convert sterol derivatives, such as cholecalciferol (vitamin D_3_) into pre-hormone 25-OH vitamin D (25(OH)D) (Bikle, 2014). DBP is also localized to the pancreatic islets. Recent studies have shown that *GC/Gc* is highly expressed in purified mouse and human α cells (Ackermann et al., 2016, Adriaenssens et al., 2016, Cigliola et al., 2018, Qiu et al., 2017, Segerstolpe et al., 2016) and is upregulated in de-differentiated β cells (Kuo et al., 2019). Because the *GC* promoter region contains cell-type-selective open chromatin regions, *GC* can be classified as an α cell signature gene, similarly to prototypical hits, such as *ARX*, glucagon (*GCG*), *IRX2*, and *DPP4* (Ackermann et al., 2016, Lam et al., 2019). Despite these findings, the role of DBP in the regulation of islet function and glucagon release remains enigmatic.

Evidence that the effects of DBP in α cells are unrelated to serum vitamin D transport comes from studies in vitamin-D-deficient patients who show no improvement in insulin-induced glucagon output upon vitamin D repletion (Gedik and Akalin, 1986). Moreover, a patient harboring a rare mutation in *GC* showed no symptoms of vitamin D deficiency, despite low plasma levels of 25(OH)D, arguing that the free form of 25(OH)D dictates many of the non-classical actions of vitamin D (Chun et al., 2014, Henderson et al., 2019). Alongside its role in 25(OH)D transport, DBP is also a major actin scavenger (Harper et al., 1987). Following disassembly of polymerized F-actin by gelsolin, DBP traps monomeric filaments using its three domains as a clamp (Otterbein et al., 2002). Pertinently, ephrin-A forward signaling has been shown to inhibit glucagon secretion through increases in F-actin density (Hutchens and Piston, 2015), and the appearance of regulated glucagon secretion in re-aggregated islets coincides with normalization of F-actin levels (Reissaus and Piston, 2017).

Linking DBP with type 2 diabetes (T2D) risk, *GC* variants are associated with elevations in fasting glucose, fasting insulin levels, and impaired responses to oral glucose challenge (Baier et al., 1998, Hirai et al., 2000, Iyengar et al., 1989, Szathmary, 1987). Results, however, tend to be conflicting, likely reflecting heterogeneity introduced by ethnicity and environment (Malik et al., 2013, Wang et al., 2014). The concept that DBP might also be involved in type 1 diabetes (T1D) risk is supported by retrospective cross-sectional analysis of 472 individuals showing that serum DBP levels were lowest in patients with T1D (Blanton et al., 2011). Using gene-expression-based genome-wide association studies, DBP was subsequently identified as a novel T1D autoantigen (Kodama et al., 2016). The same authors showed that T cell reactivity against DBP was increased in non-obese diabetic mice and that humans with T1D possess specific DBP autoantibodies (Kodama et al., 2016). Together, these studies suggest that DBP is likely to be associated with altered diabetes risk in humans.

Here, we sought to establish the role of DBP in α cell phenotype, function, and diabetes risk by combining studies in knockout mice with immunostaining analysis of pancreata from T1D donors and age-matched controls. We show that DBP contributes to proper α cell function and glucagon secretion, with related effects for δ cell morphology and insulin release. We further show that glucagon and DBP expression decrease in α cells of individuals with late-onset or long-standing T1D, but not in those with early-onset disease. As such, DBP should be considered as an essential component of the α cell and the wider islet functional machinery with relevance for glucagon secretion during diabetes.

## Results

### DBP Is Deleted in α Cells of DBP^−/−^ Mice

Mice possessing floxed *Gc* alleles do not exist, so we instead turned to a well-validated global DBP^−/−^ knockout model (Safadi et al., 1999). Given the localization of DBP to α cells and liver, as well as the existence of a patient with a loss-of-function DBP mutation (Henderson et al., 2019), we reasoned that the global DBP^−/−^ knockout model would be most appropriate for our purposes.

**Figure 1:**
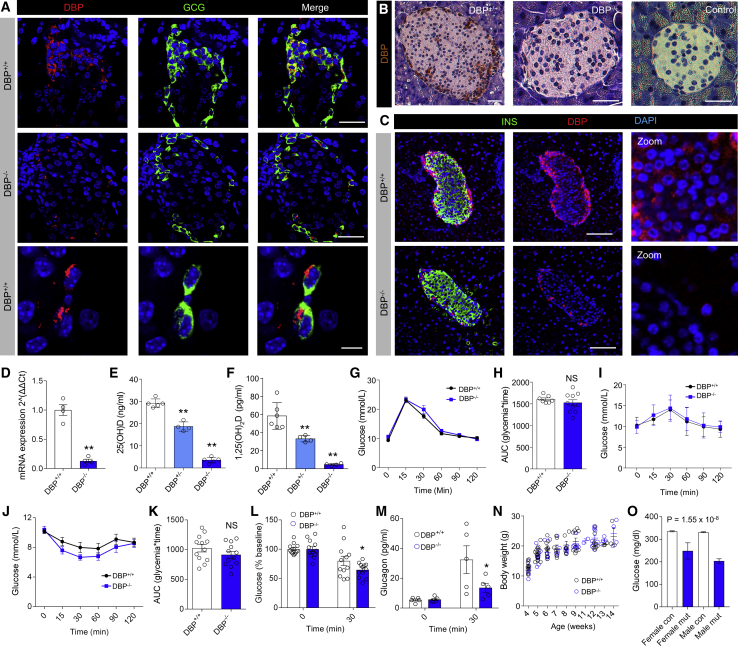
Phenotypic Assessment of DBP^−/−^ Mice (A) Representative fluorescence immunohistochemistry image showing localization of DBP to α cells and its specific loss in DBP^−/−^ animals (scale bar represents 34 μm, top and middle panels; scale bar represents 12 μm, bottom panel; n = 3 to 4 animals). (B) As for (A), but non-fluorescent DAB staining in DBP^+/+^ and DBP^−/−^ pancreatic sections (scale bar represents 40 μm; the middle and last panel are magnified relative to the first panel; n = 2 to 3 animals). (C) Fluorescence immunohistochemistry showing DBP staining of β cells, which is absent in pancreatic sections from DBP^−/−^ animals. Due to the relative strength of DBP expression in α cells, the images have been overexposed to allow visualization of DBP in the non-α-cell compartment (representative images are shown; scale bar represents 85 μm; n = 13 islets, 3 animals). (D) Expression of *Gc*, which encodes DBP, is barely detectable in DBP^−/−^ islets using Taqman assays (n = 4 to 5 animals). (E and F) Serum 25(OH)D (E) and 1,25(OH)2D (F) levels are ∼2-fold and 4-fold lowered in DBP^+/−^ and DBP^−/−^ animals, respectively (n = 4–6 animals; one-way ANOVA with Bonferroni’s multiple comparisons test). (G and H) Glucose tolerance curves (G) and area under the curve (AUC) (H) are similar in DBP^+/+^ and DBP^−/−^ mice (n = 7–11 animals; two-way repeated-measure ANOVA with Bonferroni’s multiple comparison test or unpaired t test). (I) Pyruvate tolerance is similar in DBP^+/+^ and DBP^−/−^ mice (n = 3 to 4; two-way repeated-measure ANOVA with Bonferroni’s multiple comparison test or unpaired t test). (J and K) Insulin sensitivity tends to be increased in DBP^−/−^ versus DBP^+/+^ mice (J), as also shown by the AUC (K) (n = 12 animals; two-way repeated-measure ANOVA with Bonferroni’s multiple comparisons test or unpaired t test). (L) % decrease in glucose is greater in DBP^−/−^ versus DBP^+/+^ mice 30 min post-insulin injection (n = 12 animals; two-way ANOVA with Fisher’s least significant difference [LSD]). (M) Glucagon secretion in response to insulin bolus is impaired in DBP^−/−^ mice (n = 5 animals; two-way ANOVA with Bonferroni’s multiple comparisons test). (N) Body weight/growth curve is similar in DBP^+/+^ and DBP^−/−^ mice (n = 3 to 4; two-way repeated-measure ANOVA with Bonferroni’s multiple comparison test or unpaired t test). (O) Gc^tm1.1(KOMP)Vlcg^ mice with homozygous loss of *Gc* (mut) present with decreases in fed blood glucose compared to controls (con) (n = 7 homozygous *Gc*-null males and 6 *Gc*-null females versus 666 male and 654 female controls; Mixed Model framework, linear mixed-effects model, equation without weight; both sexes classified equally). Data were obtained from the International Mouse Phenotyping Consortium (https://www.mousephenotype.org; Dickinson et al., 2016), MGI ID = MGI:95669, data release 11. Bar and line graphs show mean ± SEM. ^∗^p < 0.05, ^∗∗^p < 0.01, and NS, non-significant. DAB, 3,3′-diaminobenzidine; DBP, vitamin D-binding protein; GCG, glucagon.

Confocal imaging showed an intense DBP signal localized predominantly to GCG^+^ cells at the islet periphery in mice (Figure 1A). Although DBP expression was clearly decreased in DBP^−/−^ animals (% area DBP expression = 10.75% ± 2.05% versus 1.60% ± 0.36%, DBP^+/+^ versus DBP^−/−^, respectively; p < 0.01; Mann-Whitney test), a very faint signal could still be detected in the cytoplasm of some α cells using fluorescent immunohistochemistry (Figure 1A). This likely reflects the sensitivity of the fluorescent staining rather than antibody specificity, because we could not detect any DBP signal in DBP^−/−^ islets with the same antibody using an enzymatically amplified chromogenic staining (Figure 1B). We wondered whether DBP was also expressed in other islet cell types but might be obscured by the strong staining detected in α cells. Therefore, immunostaining was repeated using a higher antibody concentration combined with more sensitive imaging settings to oversaturate signal in α cells, but not in other cells. Using this approach, weak DBP expression could be detected in the β cell compartment, which was absent in islets from DBP^−/−^ mice (Figure 1C).

*Gc* expression was found to be ∼80% lower in DBP^−/−^ islets using specific Taqman assays (Figure 1D), and circulating 25(OH)D and hormonal 1,25-(OH)_2_ vitamin D (1,25(OH)_2_D) levels were ∼50% decreased in heterozygous DBP^+/−^ mice and almost at the limit of detection in homozygous DBP^−/−^ littermates (Figures 1E and 1F). Despite low levels of 25(OH)D and 1,25(OH)_2_D, DBP^−/−^ animals did not show signs of vitamin D deficiency unless placed on a vitamin-D-deficient diet (Safadi et al., 1999). Altogether, we can be confident that DBP is deleted in our cohort of DBP^−/−^ animals.

### DBP^−/−^ Mice Secrete Less Glucagon

Metabolic phenotyping of 8- to 12-week-old DBP^−/−^ mice revealed normal glucose tolerance (Figures 1G and 1H) and normal pyruvate tolerance versus littermate controls (Figure 1I). However, DBP^−/−^ animals tended to possess improved insulin sensitivity (Figures 1J and 1K), which was significant when the percentage change versus baseline was considered at 30 min (Figure 1L). These changes in insulin tolerance were accompanied by robust decreases in glucagon secretion in DBP^−/−^ versus DBP^+/+^ littermates (Figure 1M). Growth curves and adult body weight were similar between genotypes (Figure 1N). We next interrogated International Mouse Phenotyping Consortium data pertaining to Gc^tm1.1(KOMP)Vlcg^ mice, which also harbor whole-body deletion of DBP (MGI:5577272). Notably, mutant Gc^tm1.1(KOMP)Vlcg^ mice showed highly significant decreases in fed blood glucose (Figure 1O), consistent with lowered circulating glucagon (Brand et al., 1995).

Thus, DBP^−/−^ mice are more insulin sensitive and secrete less glucagon than their DBP^+/+^ littermates. To confirm whether these changes were associated with impaired α cell function, we proceeded to conduct the remainder of the studies in isolated pancreata and islets.

### Deletion of DBP Leads to Abnormal Islet Morphology

**Figure 2:**
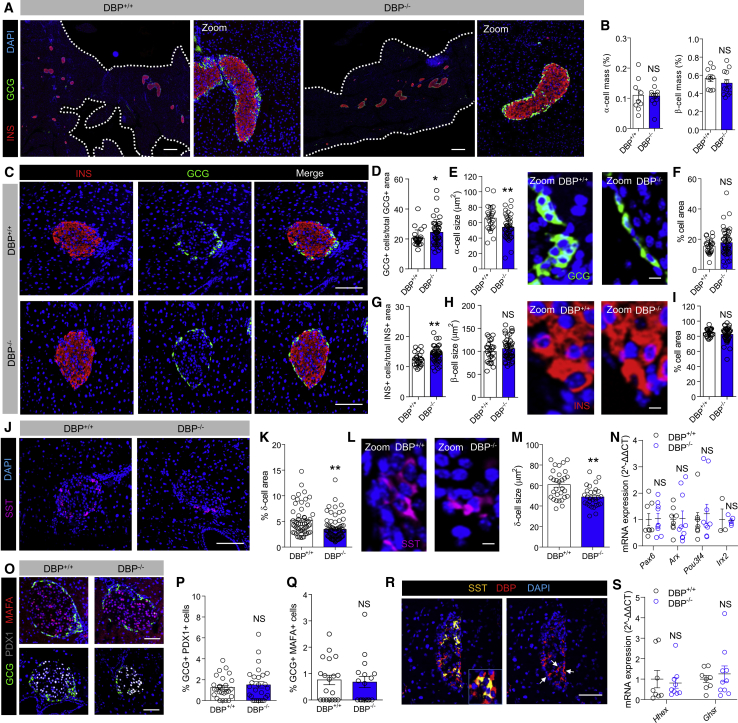
DBP Alters α Cell and δ Cell Number and Size (A and B) Cell resolution reconstruction of pancreatic sections reveals no differences in α cell and β cell mass in DBP^+/+^ and DBP^−/−^ mice (A), quantified in bar graph (B) (scale bar represents 530 μm; representative images are shown; inset is a zoom showing maintenance of cellular resolution in a single image; n = 9–12 sections from 3 to 4 animals; unpaired t test). (C–I) Morphological analyses of DBP^−/−^ islets (C) reveal increased α cell number (D) and decreased α cell size (E) (representative images shown in right panel), but normal area occupied (F). By contrast, β cell number is increased (G), although β cell size (H) (representative images shown in right panel) and area (I) are unchanged (scale bar in D represents 85 μm; scale bars in E and H represent 10 μm; n = 24–45 islets from 3 to 4 animals; E and H are zooms of C to better show α cell and β cell size; DAPI is shown in blue; unpaired t test). (J–M) δ cell proportion (J and K; n = 55–79 islets from 4 to 5 animals) and size (L and M; n = 29 to 30 islets from 3 animals) are decreased in DBP^−/−^ islets (scale bar in J represents 85 μm; scale bar in L represents 10 μm; representative images are shown; L is a zoom of J to better show δ cell size; unpaired t test). (N) Expression levels of the α cell differentiation markers *Arx*, *Pax6*, *Pou3f4*, and *Irx2* are similar in DBP^+/−^ and DBP^−/−^ islets (n = 3–10 animals; Mann-Whitney test). Note that *Arx*, *Pax6*, and *Pou3f4* were quantified using SYBR Green chemistry, whereas *Irx2* was quantified using Taqman reagents. For the sake of clarity, all genes are presented on the same graph normalized to their respective housekeeping gene. (O–Q) No changes in the proportion of α cells expressing PDX1 (O and P) or MAFA (O and Q) are detected in pancreatic sections from DBP^−/−^ versus DBP^+/+^ islets (scale bar represents 85 μm; representative images are shown; n = 17–27 islets from 3 animals; unpaired t test). (R) DBP is expressed in a subpopulation of δ cells (arrows show SST^+^/DBP^+^ cells; n = 3 animals; scale bar represents 85 μm). (S) The δ cell markers *Hhex* and *Ghsr* are not significantly different in DBP^−/−^ islets (n = 8–10 animals; one-way ANOVA with Bonferroni’s multiple comparisons test). Bar graphs show scatterplot with mean ± SEM. ^∗^p < 0.05 and ^∗∗^p < 0.01. INS, insulin; SST, somatostatin. See also Figure S1.

Cell resolution immunostaining of entire pancreatic sections showed no changes in α cell or β cell mass following loss of DBP (Figures 2A and 2B). Although detailed morphometric analyses of individual DBP^−/−^ islets revealed an increase in α cell number (Figures 2C and 2D), this was accompanied by a decrease in α cell size (Figure 2E), maintaining the area occupied by α cells (Figure 2F). While a small but significant increase in β cell number was apparent (Figure 2G), no changes in β cell size (Figure 2H) or area occupied by β cells (Figure 2I) were detected between DBP^−/−^ and DBP^+/+^ animals. However, a ∼50% decrease in δ cell mass was detected (Figures 2J and 2K), along with a reduction in the size of individual δ cells (Figures 2L and 2M).

Suggesting that loss of DBP is not associated with α cell de-differentiation, mRNA levels for *Pax6*, *Arx*, *Pou3f4*, and *Irx2* were similar in islets from DBP^+/+^ and DBP^−/−^ mice (Figure 2N). No differences in the number of (very rare) cells co-staining for GCG/PDX1 or GCG/MAFA were detected (Figures 2O–2Q), implying that adoption of a β-cell- or δ-cell-like fate by α cells was unlikely. Although DBP was expressed in some, but not all, δ cells (37.8% ± 12.9%; mean ± SD; Figure 2R), expression of the δ cell markers *Hhex* and *Ghsr* was unchanged in DBP^−/−^ islets (Figure 2S). Lastly, a similar number of proliferating cell nuclear antigen (PCNA)^+^ or proliferative α cells was detected in DBP^−/−^ and DBP^+/+^ animals, suggesting normal cell turnover rates (Figure S1).

Thus, DBP^−/−^ islets are morphologically abnormal, containing smaller and more abundant α cells alongside a modest contraction of the δ cell compartment.

### DBP Contributes to α Cell, δ Cell, and β Cell Function

**Figure 3:**
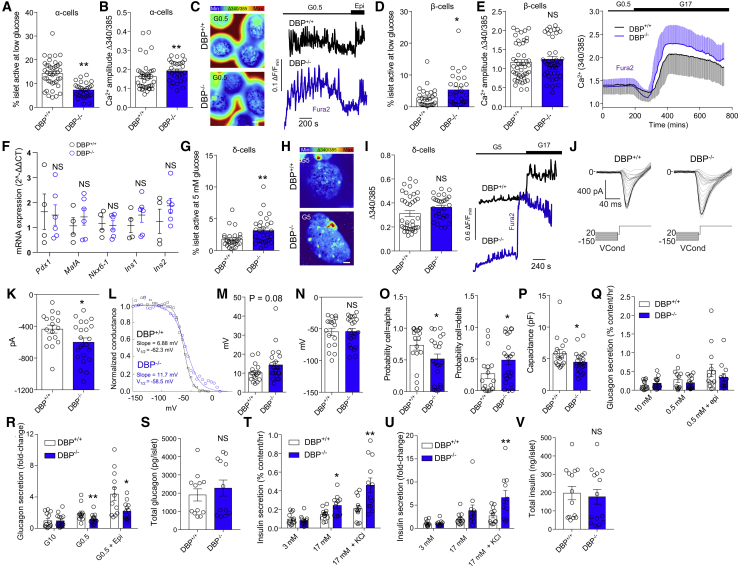
Dysregulated α cell, β cell, and δ cell Function in Islets Lacking DBP (A and B) Proportion of α cells showing activityat low (0.5 mM) glucose is decreased in DBP^−/−^ islets (A), although Ca^2+^ amplitude is increased in responsive cells (B) (n = 46–30 islets, 4 animals; Mann-Whitney test). (C) Representative images and traces showing loss of α cell activation in DBP^−/−^ islets (scale bar represents 40 μm; n = 46–30 islets, 4 animals). (D and E) Proportion of β cells showing activity at low (0.5 mM; D) glucose is increased in DBP^−/−^ islets, despite intact responses to high (17 mM) glucose (E), shown by bar graph and traces (n = 27–29 islets, 4 animals; unpaired t test). Note that error bars (SD) are shown above (DBP^−/−^) and below (DBP^+/+^) traces so as not to obscure the mean. (F) Expression of *Pdx1*, *Mafa*, *Nkx6-1*, *Ins1*, and *Ins2* is not significantly different between DBP^−/−^ and DBP^+/+^ islets (4–6 animals; paired t test). (G–I) More δ cells are active at 5 mM glucose in DBP^−/−^ compared to DBP^+/+^ islets (G and H), mounting Ca^2+^ spikes with a tendency toward increased amplitude (I) (representative Ca^2+^ images and traces are shown; images have been cropped to show a single islet; scale bar represents 40 μm; n = 28 to 29 islets, 4 animals; unpaired t test). (J and K) Representative patch-clamp recordings of α cells (J) showing increased Na^+^ conductance in DBP^-^^/^^-^ islets (K) (n = 17–22 cells, 3 animals; unpaired t test). (L–N) Sigmoid plots of raw current data showing calculation of slope factor and half-maximal voltage (V_1/2_) for two cells (L). Summary data show a tendency toward increased slope factor for Na^+^ channel inactivation (M) but unchanged half-maximal voltage (N) in DBP^−/−^ versus DBP^+/+^ α cells (n = 17–22 cells, 3 animals; Mann-Whitney test). (O) Electrophysiological fingerprinting reveals decreased and increased probability of cells resembling an α cell or δ cell, respectively, in DBP^−/−^ islets (n = 17–22 cells, 3 animals; Mann-Whitney test). (P) α cell capacitance is significantly reduced in DBP^−/−^ islets (n = 17–22 cells, 3 animals; unpaired t test). (Q and R) Glucagon secretion is impaired in DBP^−/−^ islets in response to low (0.5 mM) glucose and low (0.5 mM) glucose + epinephrine, shown normalized to content (Q) or fold-change (R) (n = 12–14 replicates, 10 animals; two-way ANOVA with Bonferroni’s multiple comparisons test or Mann-Whitney test). (S) Glucagon content is similar in DBP^+/+^ and DBP^−/−^ islets (n = 12 replicates, 8 animals; unpaired t test). (T and U) Insulin secretion in response to high (17 mM) glucose or high (17 mM) glucose + KCl is increased in DBP^−/−^ islets, shown normalized to content (T) or fold change (U) (n = 11–13 replicates, 3 animals; two-way ANOVA with Bonferroni’s multiple comparisons test or unpaired t test). (V) Insulin content is similar in DBP^+/+^ and DBP^−/−^ islets (n = 14 replicates, 3 animals; unpaired t test). Bar graphs show scatterplot with mean ± SEM. Traces show mean ± SD. ^∗^p < 0.05 and ^∗∗^p < 0.01.

Multicellular Ca^2+^ imaging of DBP^−/−^ islets showed large impairments in the activity of α cells, identified by their responses to low glucose (0.5 mM) and epinephrine (Figures 3A–3C) or silencing at high (17 mM) glucose (Tian et al., 2011). This presented as a loss of α cell activation by low glucose (Figure 3A; Videos S1 and S2), although some α cells that remained active displayed characteristic Ca^2+^-spiking responses of elevated amplitude (Figures 3B and 3C).

Video S1. Ca^2+^ Recording (Fura2) in a DBP^+/+^ Islet at 0.5 mM Glucose Followed by Application of 0.5 μM Epinephrine (a Single Cropped Islet Is Shown; Playback = 40 Frames per Second), Related to Figure 3

Video S2. Ca^2+^ Recording (Fura2) in a DBP^−/−^ Islet at 0.5 mM Glucose Followed by Application of 0.5 μM Epinephrine (a Single Cropped Islet Is Shown; Playback = 40 Frames per Second), Related to Figure 3

The same islets were also examined for changes in β cell activity at both low (0.5 mM) and high (17 mM) glucose. A large increase in the proportion of β cells active at low glucose was observed (Figure 3D), identified on the basis of their responsiveness to subsequent challenge with high glucose. However, no differences in β cell activity were detected at high glucose (Figure 3E), suggesting the presence of intact glucose metabolism. Gene expression analyses showed no significant changes in levels of the β cell transcription factors *Pdx1*, *Mafa*, *Nkx6-1*, as well as *Ins1* and *Ins2* (Figure 3F).

To record δ cell activity, islets were imaged at 5 mM glucose before increasing concentration of the sugar to 17 mM. δ cells are identified by their characteristic, rare, Ca^2+^-spiking patterns at 5 mM glucose, which are maintained in the presence of high glucose (compared to β cells and α cells that are inactive at 5 mM and 17 mM, respectively; Shuai et al., 2016, Vierra et al., 2018). Suggesting the presence of abnormal function, the proportion of active δ cells was increased in DBP^−/−^ islets (Figures 3G and 3H; Videos S3 and S4), possibly reflecting compensation for reduced δ cell number. Responsive δ cells displayed Ca^2+^ spikes of normal amplitude (Figure 3I). Although the number of α cells active at low (0.5 mM) glucose was also increased in DBP^−/−^ islets, these cells would be silent at high glucose, thus allowing their differentiation from δ cells.

Video S3. Ca^2+^ Recording (Fura2) in a DBP^+/+^ Islet at 5 mM Glucose Followed by Transition to 17 mM Glucose (a Single Cropped Islet Is Shown; Playback = 40 Frames per Second), Related to Figure 3

Video S4. Ca^2+^ Recording (Fura2) in a DBP^−/−^ Islet at 5 mM Glucose Followed by Transition to 17 mM Glucose (a Single Cropped Islet Is Shown; Playback = 40 Frames per Second), Related to Figure 3

### DBP Is Required for Normal α Cell Na^+^ Channel Conductance

As part of α cell electrical activity, Na^+^ channel inactivation properties play an important role in determining glucagon secretion (Zhang et al., 2013). Using patch-clamp electrophysiology, we therefore explored whether DBP influences α cell Na^+^ channel function. As expected from the increased Ca^2+^ amplitude detected in these cells, Na^+^ currents were increased in α cells lacking DBP (Figures 3J and 3K). The slope factor of Na^+^ inactivation also tended to be increased (Figures 3L and 3M), despite a similar half-maximal voltage (Figure 3N), suggesting the presence of impairments in glucose-dependent α cell activity.

We further subjected patch-clamp recordings from DBP^+/+^ and DBP^−/−^ islets to a mathematical model (Briant et al., 2017). This model takes as input the electrophysiological data of a cell and outputs a probability that the cell is an α cell. Although the model predicted α cell phenotype in DBP^+/+^ islets with high probability, confidence was lower in recordings from DBP^−/−^ islets with the output favoring a more δ-cell-like (probability = 0.27 versus 0.48, DBP^+/+^ versus DBP^−/−^, respectively; p < 0.05), but not β-cell-like (probability = 4.43 × 10^−7^ versus 1.15 × 10^−8^, DBP^+/+^ versus DBP^−/−^, respectively; non-significant), profile (Figure 3O). Thus, α cells lose their “electrophysiological identity,” become less α cell like, and resemble δ cells following loss of DBP. This alteration in phenotype would not be expected to interfere with the identification of α cells using Ca^2+^ imaging, because epinephrine was used to differentiate α cells, β cells, and δ cells. In any case, the change in proportion of cells active at low glucose supports the finding here that α cells lose their phenotype. Confirming the decrease in α cell size detected using immunohistochemistry, membrane capacitance was significantly lower in cells predicted to be α cells in DBP^−/−^ islets (Figure 3P).

### DBP Regulates Glucagon and Insulin Secretion

DBP^+/+^ islets responded to low (0.5 mM) glucose with a 2-fold increase in glucagon secretion (Figures 3Q and 3R). By contrast, DBP^−/−^ islets showed a tendency toward increased basal glucagon levels and loss of glucagon secretion in response to low glucose (Figures 3Q and 3R). Glucagon secretory responses to epinephrine were also significantly impaired in DBP^−/−^ islets, pointing toward a defect in either adrenergic receptor signaling or exocytosis (Figures 3Q and 3R). This defect is unlikely to be associated with altered electrical signaling, because epinephrine does not influence membrane potential (Hamilton et al., 2018). Suggesting the presence of normal glucagon biosynthesis, total levels of the hormone were similar between DBP^+/+^ and DBP^−/−^ littermates (Figure 3S).

Conversely to glucagon, glucose-stimulated insulin secretion was significantly increased in islets from DBP^−/−^ animals (Figures 3T and 3U). This effect was likely associated with improved exocytosis of the readily releasable pool of insulin granules, because KCl-stimulated insulin secretion was significantly higher in DBP^−/−^ versus DBP^+/+^ islets (Figures 3T and 3U), despite equivalent insulin content (Figure 3V).

Thus, DBP is required for normal glucose-regulated glucagon secretion and limits insulin secretion under high-glucose stimulation.

### DBP Mediates α Cell and β Cell Function through F-actin Binding

**Figure 4:**
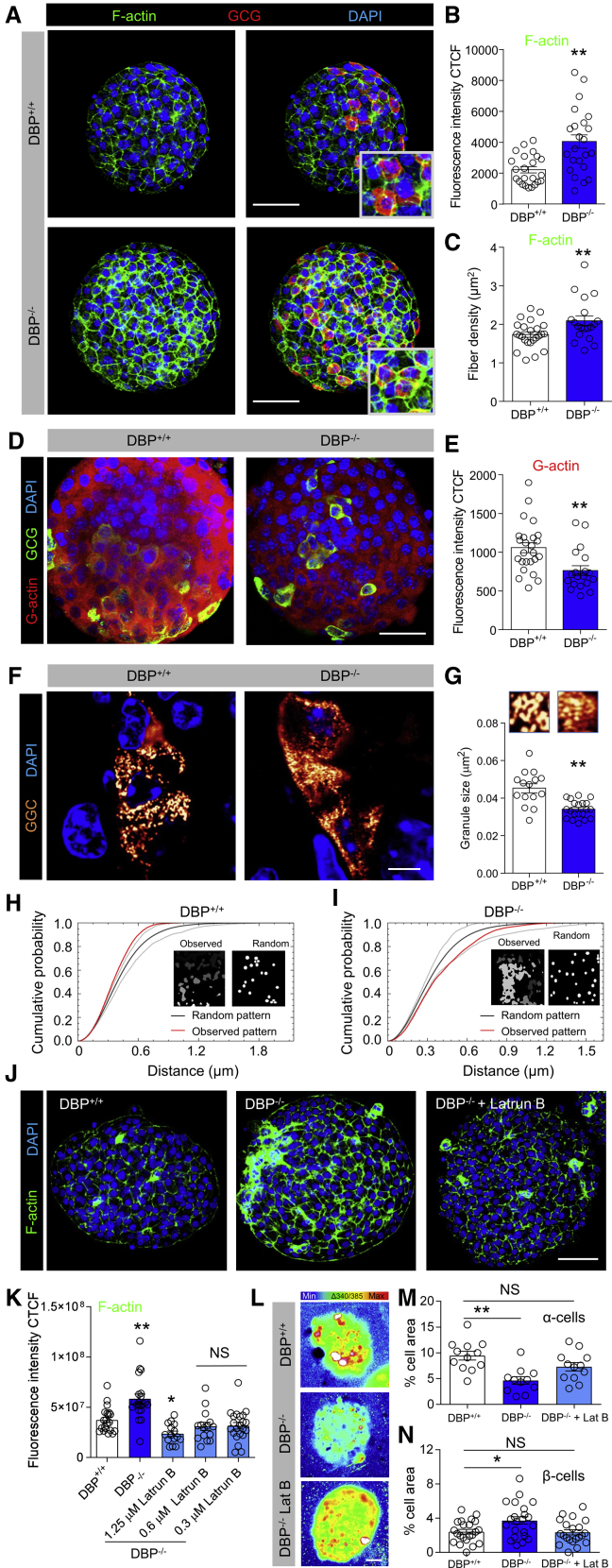
DBP Scavenges Actin in the Islet and Maintains Glucagon Granule Morphology (A–C) F-actin abundance is increased following loss of DBP (A), quantified using fluorescence intensity (B) and fiber density (C) (representative images are shown; scale bar represents 53 μm; n = 23 islets, 3 animals; unpaired t test). (D and E) As for (A)–(C) but representative images (D) and summary bar graph (E) showing decreased G-actin monomer expression in DBP^−/−^ islets (scale bar represents 34 μm; n = 19–25 islets, 5 animals; unpaired t test). (F and G) Representative super-resolution Airyscan (∼140 nm lateral resolution) snapshots of glucagon granules (F), showing an ∼20% decrease in size (G) (magnified images from F are shown above each bar; scale bar represents 6 μm; n = 13–15 islets, 3 to 4 animals; unpaired t test). (H and I) Representative G-function analysis on actual and simulated glucagon granule distribution showing that glucagon granules tend to be more clustered in DBP^+^^/^^+^ (H) compared to DBP^−/−^ (I) islets (actual and simulated distribution is inset). (J and K) Representative images (J) and bar graph (K) showing that F-actin levels are increased in DBP^−/−^ islets but can be restored to almost DBP^+/+^ levels using 0.3–1.25 μM Latrunculin B (scale bar represents 53 μm; n = 15–22 islets, 3 to 4 animals; one-way ANOVA with Dunnett’s post hoc test). (L and M) Representative images (L) and summary bar graph (M) showing that 0.3 μM Latrunculin B rescues α cell responses to low (0.5 mM) glucose in DBP^−/−^ islets (images have been cropped to show a single islet; scale bar represents 25 μm; n = 11 to 12 islets, 3 to 4 animals; one-way ANOVA with Tukey’s post hoc test). (N) Latrunculin B (0.3 μM) rescues basal (3 mM glucose) β cell Ca^2+^ activity in DBP^−/−^ islets (n = 21 to 22 islets, 5 animals; one-way ANOVA with Tukey’s post hoc test). Bar graphs show scatterplot with mean ± SEM. ^∗^p < 0.05 and ^∗∗^p < 0.01. Latrun B or Lat B, Latrunculin B.

DBP is a major actin scavenger and might exert effects on α cell size and glucagon secretion by trapping monomeric actin (G-actin), which is needed to form polymerized actin (F-actin; Dominguez and Holmes, 2011). To investigate DBP-actin interactions, high-resolution snapshots were taken of islets stained with either phalloidin or DNAaseI to demarcate F-actin and G-actin, respectively. Increases in F-actin staining intensity and fiber density were seen throughout DBP^−/−^ islets (Figures 4A–4C), rather than restricted solely to α cells. Conversely, G-actin levels were reduced in DBP^−/−^ mice, again being evident throughout the islet (Figures 4D and 4E).

Because F-actin/G-actin ratio is important for regulated secretion (Kalwat and Thurmond, 2013), glucagon granule size and distribution were mapped in pancreatic slices using super-resolution imaging. Analysis of individual granules revealed a small but significant decrease in granule size in DBP^−/−^ mice, although area occupied was unchanged, pointing to an increase in granule number (Figures 4F and 4G). Although glucagon granules tended to be clustered in DBP^+/+^ α cells, they were more diffusely scattered throughout the cytoplasm in DBP^−/−^ tissue (Figures 4H and 4I). We note that, at the lateral resolutions achieved here (140 nm), measurement of individual glucagon granules (200–400 nm; Pfeifer et al., 2015) is possible. We concede that electron microscopy would be needed to definitively measure glucagon granule size and morphology, although other issues become problematic with this technique, such as dehydration artifacts.

Lastly, we investigated whether normal activity could be rescued in DBP^−/−^ islets by reducing F-actin levels to DBP^+/+^ levels. Experiments were performed in the absence or presence of Latrunculin B, which prevents F-actin polymerization (Spector et al., 1983). A concentration response showed that Latrunculin B was able to modulate F-actin within the range detected in DBP^+/+^ islets (Figure 4J). Notably, by using 0.3 μM Latrunculin B to reduce F-actin in DBP^−/−^ islets down to DBP^+/+^ levels (Figure 4K), we were able to partially restore both α cell and β cell Ca^2+^ responses to low glucose (Figures 4L–4N).

Together, these results suggest that DBP knockout increases availability of monomeric G-actin, which is then able to polymerize to form F-actin, ultimately altering granule distribution and size, as well as α cell function.

### DBP and Glucagon Expression Is Decreased in Late-Onset and Long-Standing T1D Donors

**Figure 5:**
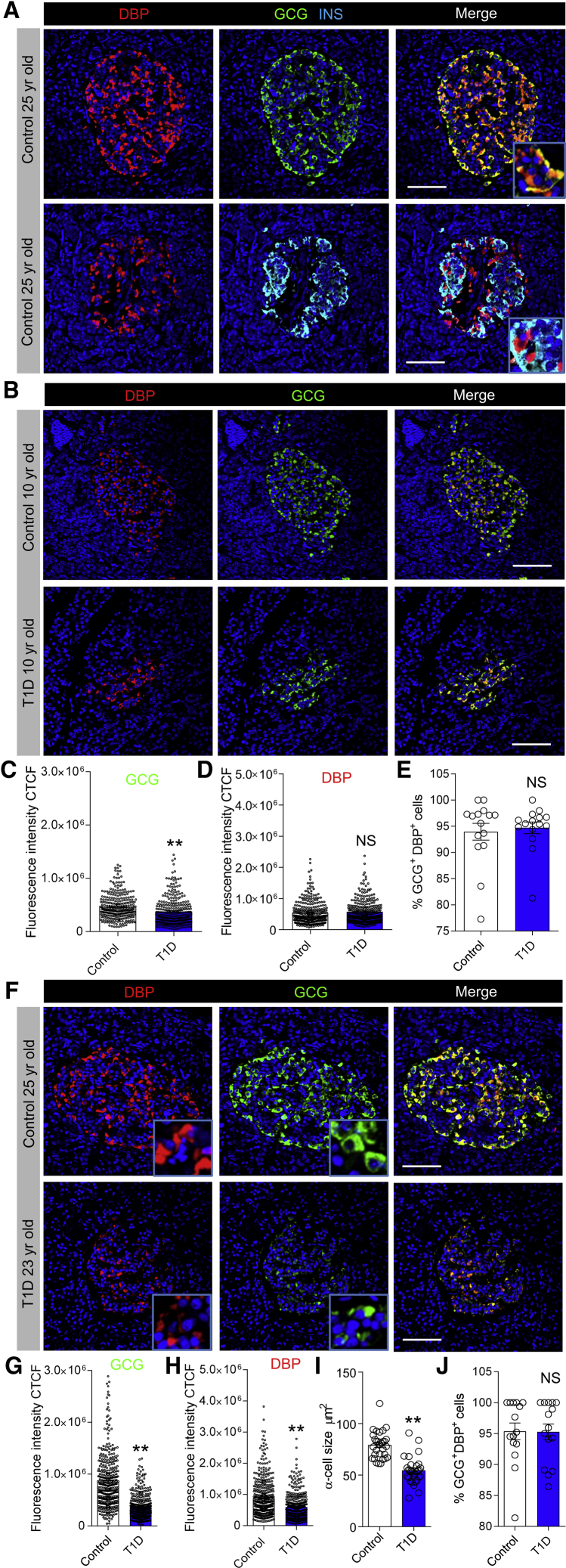
DBP Expression Is Decreased in Late-Onset and Long-Standing Type 1 Diabetes (A) Fluorescent immunohistochemistry showing strong expression of DBP in the α cell compartment in human islets (inset shows a zoomed image; n = 7 control donors). (B–E) Glucagon staining decreases slightly (B and C) in islets of donors with early-onset (≤10 years old) T1D, but this is not associated with changes in DBP expression (B and D) or proportion of DBP^+^/GCG^+^ α cells (E) (representative images are shown; inset shows a zoomed image; n = 300 cells, 30 islets, 3 T1D donors and age-matched controls; from the Exeter biobank; Mann-Whitney test). (F–H) Glucagon (F and G) and DBP (F and H) expression are both decreased in islets of donors with late-onset (≥15 years old) T1D (representative images are shown; inset shows a zoomed image; n = 400 cells, 40 islets, 4 T1D donors and age-matched controls; Mann-Whitney test). (I and J) α cell size (I), but not proportion of DBP^+^/GCG^+^ α cells (J), is decreased in islets of donors with late-onset (≥15 years old) T1D (n = 180 cells, 30 islets, 3 T1D donors and age-matched controls; inset shows a zoomed image; unpaired t test). Scale bar represents 42.5 μm. Bar graphs show scatterplot with mean ± SEM. ^∗∗^p < 0.01. See also Figures S2–S4 and Table S1.

Islet α cells persist in T1D but display reduced glucose responsiveness (Brissova et al., 2018, Gerich et al., 1973), which could be associated with altered DBP expression. We therefore examined whether DBP levels changed in T1D, initially using pancreatic sections from the Exeter Archival Diabetes Biobank. Immunohistochemistry was performed on sections from donors with early- (≤10 years old) and late-onset (≥15 years old) T1D, together with their age-matched controls. In donors without diabetes, DBP was highly localized to α cells (Figure 5A), as previously shown (Kodama et al., 2016, Lam et al., 2019), although we were also able to detect very faint expression in β cells, as for mouse (Figure S2). In sections from donors with T1D, a similar pattern of DBP immunostaining was observed (Figure 5B). Although a small but significant decrease in glucagon expression was seen in islets of early-onset T1D donors (Figure 5C), this was not accompanied by changes in DBP staining (Figure 5D) or proportion of α cells immunopositive for DBP (Figure 5E).

By contrast, glucagon levels were almost 2-fold lower in islets of late-onset T1D donors versus age-matched controls (Figures 5F and 5G), in line with previous reports of decreased α cell mass during T1D (Bonnet-Serrano et al., 2018). These changes were accompanied by a reduction in DBP expression (Figure 5H) and α cell size (Figure 5I), although no differences were detected in the number of DBP^+^/GCG^+^ cells per islet (Figure 5J). We were able to confirm results in samples from IsletCore (Alberta) and also show that DBP levels consistently decrease in islets of donors with more long-standing T1D (Figure S3).

**Figure 6:**
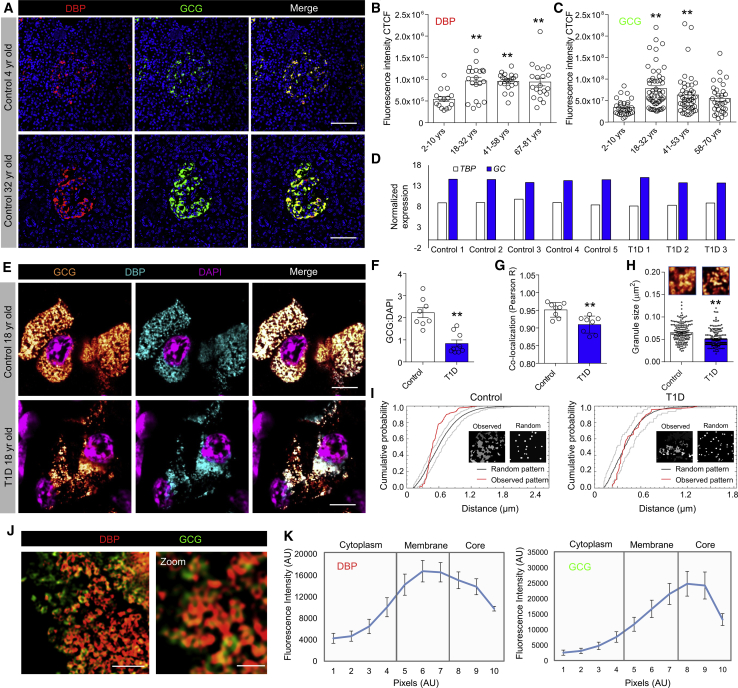
DBP Expression Increases with Age and Co-localizes with Glucagon in Granules (A–C) DBP (A and B) and glucagon (A and C) expression increase with age in control donors (representative images are shown; scale bar represents 42.5 μm; n = 15–53 islets per age group, 3 early-onset and 4 late-onset T1D donors together with age-matched controls; one-way ANOVA with Tukey’s multiple comparison test). (D) Analysis of a published RNA-seq dataset from purified α cells (Brissova et al., 2018) shows no difference in transcript abundance for *GC* (encoding DBP) in control and T1D donors. Expression levels are normalized against *TBP*. Each individual donor is shown. Data were obtained from GEO: GSE106148. (E) Super-resolution images showing co-localization of DBP and glucagon within the same granule in α cells of both control and late-onset T1D donors (representative images are shown; scale bar represents 6 μm; n = 8 cells from 8 islets, 4 late-onset T1D donors together with age-matched controls). (F and G) The ratio of glucagon:DAPI (F) and DBP/glucagon co-localization strength (G) is lower in α cells from donors with late-onset T1D (n = 8 cells, 4 late-onset T1D donors together with age-matched controls; unpaired t test). (H) Glucagon granule size is decreased in α cells from donors with late-onset T1D (magnified images from E are shown above each bar; n = 160–200 granules from 4 islets, 4 late-onset T1D donors together with age-matched controls; Mann-Whitney test). (I) Representative G-function analysis on actual and simulated glucagon granule distribution showing a more random arrangement of glucagon granules in α cells of T1D donors (actual and simulated distribution is inset). (J and K) Structured illumination microscopy shows DBP localized to the glucagon granule membrane (J), quantified as fluorescence intensity along a line spanning cytoplasm-granule membrane-granule core (K) (scale bar in J: left panel represents 1.5 μm, right panel represents 0.5 μm; n = 11 line profiles, 3 donors). Bar graphs show scatterplot with mean ± SEM. Line graphs shown mean ± SEM. ^∗∗^p < 0.01. TBP, TATA-box binding protein. See also Table S1.

Immunohistochemistry of control islets showed that DBP expression increased with age, peaking at 18–32 years and remaining elevated thereafter (Figures 6A and 6B). Similar results were seen for glucagon expression (Figure 6C). Glucagon and DBP expression values for each individual donor are provided in Figure S4.

### Granular DBP Content Decreases in Late-Onset T1D

Analysis of a published RNA sequencing (RNA-seq) dataset (Brissova et al., 2018) revealed no differences in *GC* expression in purified α cells from control and T1D donors (20–53 years; Figure 6D), suggesting that DBP might be post-transcriptionally regulated. As such, we investigated DBP and glucagon localization within human α cells using super-resolution microscopy. Unexpectedly, DBP was found to be present in glucagon granules of control donors (Figure 6E), suggesting that DBP enters the secretory pathway and might function in an autocrine manner.

Explaining the decrease in glucagon and DBP expression in late-onset T1D samples, a large reduction in the number of GCG^+^/DBP^+^ granules was detected in each α cell (Figure 6F), accompanied by a small decrease in DBP/GCG co-localization (Figure 6G). Glucagon granules were also smaller and more randomly distributed in late-onset T1D α cells (Figures 6H and 6I), suggestive of changes in the actin cytoskeleton, as demonstrated for mice.

Using structured illumination microscopy (SIM) (110 nm lateral resolution), we were able to observe the presence of DBP on the glucagon granule membrane (Figures 6J and 6K), further confirming the quantitative nature of our approaches and suggesting that DBP might act on the cell membrane following vesicle fusion (rather than be co-secreted with the glucagon cargo).

## Discussion

In the present study, we show that DBP is strongly expressed in murine and human α cells. Loss of DBP leads to alterations in α cell number and size, electrical activity, and glucagon release both *in vitro* and *in vivo*. This is accompanied by changes in δ cell mass, as well as alterations in β cell function and insulin release. Linking these findings, DBP was found to decrease the availability of actin monomeric subunits for assembly into polymers. DBP expression levels were also decreased in islets of donors with late-onset or long-standing T1D, but not in children with early-onset disease.

Transcriptomic studies have consistently shown that *Gc* is highly enriched in the mouse α cell lineage (Adriaenssens et al., 2016, Cigliola et al., 2018, Qiu et al., 2017), similarly to data from humans (Ackermann et al., 2016, Segerstolpe et al., 2016). In keeping with these findings, our immunohistochemical analyses confirmed that DBP is predominantly expressed at the protein level in α cells in mouse as well as human (Kodama et al., 2016, Lam et al., 2019). Careful inspection of images also detected faint DBP expression in β cells, which was difficult to appreciate due to the high intensity of the DBP signal in α cells. Notably, DBP expression was absent in β cells in DBP^−/−^ islets, and F-actin and G-actin were both altered across the islet. Thus, DBP is detectable in both α cells and β cells, with large differences in expression levels apparent between the two compartments. In addition, recent studies have shown that β cells increase histone modification at the *Gc* locus in response to high-fat feeding, with knockout of *Gc* protecting from β cell dysfunction (Kuo et al., 2019). As such, DBP protein expression in the β cell compartment is low but is likely to be upregulated under conditions of metabolic stress.

Previous studies using a KOMP mouse (Gc^tm1.1(KOMP)Vlcg^) showed that glucagon secretion was not different between islets from knockout (KO) animals and control littermates fed high-fat diet (Kuo et al., 2019). It should be noted, however, that the authors also included mice possessing 1 × wild-type *Gc* allele as controls (i.e., heterozygotes), which we show here is associated with a 50% reduction in both 25(OH)D and 1,25(OH)_2_D concentrations. Therefore, we think that the discrepancies between our studies and those of Kuo et al. (2019) likely reflect inability to detect differences between glucagon secretion in homozygous and heterozygous animals. Indeed, well-powered clinical chemistry phenotypic assays of Gc^tm1.1(KOMP)Vlcg^ mice showed significant decreases in fed blood glucose levels, which would be expected in the presence of hypoglucagonemia. Further work up of glucagon-centric measures in Gc^tm1.1(KOMP)Vlcg^ and DBP^−/−^ mice might be interesting, comparing directly homozygous, heterozygous, and wild-type littermates.

Intriguingly, Na^+^ currents, which contribute to action potential firing in α cells, were increased in DBP^−/−^ α cells. This was mirrored at the level of low glucose-stimulated Ca^2+^ rises, which were also increased by loss of DBP. Given that α cell function was otherwise decreased across the board (i.e., defective glucagon secretion, activation by low glucose, electrophysiological identity, and Na^+^ channel inactivation), increases in Na^+^ conductance and Ca^2+^ spiking amplitude are likely to reflect a maladaptive compensatory response. Alternatively, these changes could alter glucagon/glutamate feedback at high glucose (Caicedo, 2013), subsequently leading to blunted glucagon secretion at low glucose. The changes in α cell function are unlikely to be related to de-differentiation or reductions in δ cell activity, because expression of α-cell-specific transcription factors was unaffected by loss of DBP and somatostatin has been shown to inhibit glucagon secretion (Mandarino et al., 1981).

Supporting the notion that F-actin is an important regulator of glucagon release (Hutchens and Piston, 2015), fiber density was increased in DBP^−/−^ animals, alongside decreases in monomeric G-actin content. Thus, DBP in the α cell likely scavenges G-actin, preventing formation of F-actin polymers, which would otherwise suppress glucagon release. At the molecular level, F-actin has been shown to restrict basal insulin release (Kalwat and Thurmond, 2013), as well as maximal glucagon secretion, by acting as a physical constraint against granule exocytosis (Hutchens and Piston, 2015, Reissaus and Piston, 2017). Morphological evidence for this was provided in the current study by the observation that glucagon granules were distributed more diffusely in DBP^−/−^ islets. Furthermore, granule size was decreased, indicative of either sequestration of immature granules or preferential release of larger, more mature granules. Indeed, F-actin has been shown to influence granule transport and retention through its barrier and scaffold functions (Gutiérrez and Villanueva, 2018). Cytoskeletal changes were also likely to be involved in the reduction in α cell and δ cell size, because assembly of actin filaments from monomers is critical for cell morphology (Pollard and Cooper, 2009).

β cells displayed F-actin-dependent increases in Ca^2+^ activity at low glucose in DBP^−/−^ islets. This, however, did not translate to elevated basal insulin release, probably because F-actin fiber density was also increased across β cells, potentially acting as a barrier for unregulated granule exocytosis under low glucose conditions (Mziaut et al., 2016). Although DBP levels were much lower in β cells compared to α cells, it could be argued that only small amounts are required to prevent actin polymerization, given the high binding affinity for monomeric actin (Mc Leod et al., 1989). By contrast, lack of α cell shutoff at high glucose would be expected to positively influence β cell activity and increase glucose-stimulated insulin secretion (Svendsen et al., 2018). An alternative mechanism might center on the decrease in δ cell number, which would be expected to decrease the tonic negative somatostatin tone exerted on β cells (van der Meulen et al., 2015). It should be noted that the actin barrier would not feature under conditions of β cell stimulation, because glucose induces dramatic actin remodeling through gelsolin (Tomas et al., 2006), which binds actin filaments in competition with DBP. Indeed, such cytoskeletal remodeling is likely to explain some of the seemingly opposite effects of DBP loss on α cell and β cell function, because the actin-scavenging properties of DBP become relatively less important at high glucose.

Demonstrating the relevance of our studies for human disease, islets in pancreata from individuals with late-onset or long-standing T1D consistently showed decreased DBP expression, as well as a reduction in α cell size. Super-resolution imaging showed that the majority of glucagon granules in human α cells also contained DBP, with a sharp decrease in granular expression levels during T1D. A similar localization of DBP to secretory granules was reported in human neutrophils, together with release of the protein into the extracellular milieu (Kew et al., 1993). Because the *GC* transcriptional machinery is present in the α cell, the source of this DBP is likely from *de novo* synthesis. It is also plausible that DBP is transported from the circulation into α cells by megalin-mediated endocytosis, as reported in the kidney (Nykjaer et al., 1999), and that either this process or liver production of DBP is altered during T1D. However, it is difficult to envisage how endocytosis would lead to accumulation of DBP specifically on the membrane of glucagon granules.

Interestingly, no changes in DBP expression were found in early-onset T1D donors. However, DBP expression was significantly lower in young versus older control donors without T1D. These data suggest that DBP expression and thus α cell identity might not be fully specified until adolescence, meaning that DBP cannot be further downregulated in early-onset T1D. Although defects in α cell function are observed in patients with early-onset diabetes (Siafarikas et al., 2012), this presumably stems from DBP-independent mechanisms, which are then exacerbated with age as decreases in DBP become relatively more important. Indeed, α cell dysfunction during T1D is likely caused by multiple non-mutually exclusive mechanisms or insults, as for β cell failure during T2D.

These data raise a number of interesting questions involving the known role of DBP as a novel autoantigen during T1D (Kodama et al., 2016). For example, does DBP only act as an autoantigen in late-onset T1D patients? Is the decrease in DBP expression seen in late-onset T1D a consequence of autoantigens or another unrelated mechanism? If DBP is an autoantigen in T1D, why do α cells not die, or are the low DBP-expressing β cells instead targeted? Could α cells confer autoimmunity on β cells through paracrine DBP signaling? How do these findings relate to polymorphic variants in *GC*, which are known to influence DBP action/levels, as well as 25(OH)D (Powe et al., 2013)? Further systematic studies in autoantigen-positive and negative early- and late-onset T1D donors, as well as individuals harboring *GC* risk alleles, will be required to address these questions. Nonetheless, our studies suggest that, together with adoption of a β-cell-like transcriptional profile (Brissova et al., 2018), loss of DBP might contribute to the impaired glucagon secretion reported in T1D (Brissova et al., 2018, Marchetti et al., 2000).

We acknowledge a number of limitations in the present study. First, the animals were globally deleted for DBP, which means that the effects of the protein specifically in α cells could not be examined. However, DBP is highly expressed in α cells, which validates our model. Moreover, use of DBP^−/−^ mice allowed us to uncover a hitherto underappreciated role of DBP in regulating β cell and δ cell function, and a global deletion model would be more reflective of studies in humans bearing homozygous deletion of *GC* (Henderson et al., 2019). Nonetheless, it will be interesting in the future to recombine animals bearing floxed alleles with the Gcg-CreER^T2^ deleter line (Ackermann et al., 2017), which would also avoid any issues with loss of DBP during critical phases of α cell development. Second, although animals were fed a vitamin-D-sufficient diet, we cannot completely exclude vitamin-D-dependent effects of DBP. Suggesting that this is unlikely to be the case, a single individual harboring homozygous mutations in *GC* did not show symptoms consistent with vitamin D deficiency despite very low plasma 25(OH)D levels (Henderson et al., 2019). This argues for the free hormone hypothesis, where DBP acts as a major vitamin D reservoir, but only low levels are required for biological effects (Chun et al., 2014). Third, morphometric analyses were based upon glucagon staining, which could lead to an underestimation of α cell size in T1D samples, especially if the fewer detectable granules were not distributed evenly throughout the cytoplasm. Moreover, we only used samples from two biobanks, and as such, differences in sample processing and storage could contribute to the findings reported here. Fourth, although the mice did not show a clear phenotype, further studies are warranted using *in vivo* models of α cell stress, for example, glucagon receptor antagonism or high-fat diet (Gu et al., 2009, Merino et al., 2015), preferably using a conditional knockout. Lastly, although a causal role for DBP in α cell dysfunction is suggested by mouse studies, we cannot confidently assert the same in islets of human T1D donors where autoimmunity and species differences come into play.

In summary, we show that DBP contributes to α cell phenotype and glucagon secretion, with changes in expression apparent during late-onset and long-standing T1D. The stage is now set for investigating more widely how DBP influences islet function and disease risk in individuals with T1D and T2D.

## STAR★Methods

### Key Resources Table

**Table:** 

REAGENT or RESOURCE	SOURCE	IDENTIFIER
**Oligonucleotides**		

*Ppia* For: AAGACTGAGTGGTTGGATGG	Sigma-Aldrich	N/A
*Ppia* Rev: ATGGTGATCTTCTTGCTGGT		
*Pax6* For: CAGTGTCTACCAGCCAATCC	Sigma-Aldrich	N/A
*Pax6* Rev: GCACTGTACGTGTTGGTGAG		
*Arx* For: TTCCAGAAGACGCACTACCC	Sigma-Aldrich	N/A
*Arx* Rev: TCTGTCAGGTCCAGCCTCAT		
*Pou3f4* For: CCGACCAGCATTGACAAGATC	Sigma-Aldrich	N/A
*Pou3f4* Rev: GAGGTTCGCTTCTTGCGTTT		
*Irx2*	Thermo Fisher Scientific	Taqman: Mm01340316_m1
*Hhex* For: CGAGACTCAGAAATACCTCTCCC	Sigma-Aldrich	N/A
*Hhex* Rev: CTGTCCAACGCATCCTTTTTG		
*Ghsr* For: GCTCACCGTGATGGTATGGG	Eurofins	N/A
*Ghsr* Rev: CCCGATGAGACTGTAGAGCAC		
*Pdx1* For: ACTTAACCTAGGCGTCGCACAAGA	Sigma-Aldrich	N/A
*Pdx1* Rev: GGCATCAGAAGCAGCCTCAAAGTT		
*Mafa* For: CGGGAACGGTGATTGCTTAG	Sigma-Aldrich	N/A
*Mafa* Rev: GGAGGTTGGGACGCAGAA		
*Nkx6.1* For: GCCTGTACCCCCCATCAAG	Sigma-Aldrich	N/A
*Nkx6.1* Rev: GTGGGTCTGGTGTGTTTTCTCTT		
*Ins1* For: GCTGGTGGGCATCCAGTAA	Sigma-Aldrich	N/A
*Ins1* Rev: AATGACCTGCTTGCTGATGGT		
*Ins2* For: GAAGTGGAGGACCCACAAGT	Sigma-Aldrich	N/A
*Ins2* Rev: GATCTACAATGCCACGCTTC		
*Gc*	Thermo Fisher Scientific	Taqman: Mm04243540_m1
*Gusb*	Thermo Fisher Scientific	Taqman: Mm01197698_m1

**Antibodies**		

Guinea pig anti-PDX1	Abcam	Abcam Cat# ab47308; RRID:AB_777178
MAFA	Bethyl Laboratories	Bethyl Cat# IHC-00352; RRID:AB_1279486
Rabbit anti-insulin	Cell Signaling Technology	Cell Signaling Technology Cat# 3014; RRID:AB_2126503
Guinea pig anti-insulin	Abcam	Abcam Cat# ab7842; RRID:AB_306130
Mouse monoclonal anti-glucagon	Sigma-Aldrich	Sigma-Aldrich Cat# G2654; RRID:AB_259852
Rabbit anti-glucagon	Sigma-Aldrich	Sigma-Aldrich Cat# SAB4501137; RRID:AB_10761583
Mouse anti-somatostatin	Thermo Fisher Scientific	Thermo Fisher Scientific Cat# 14-9751-80; RRID:AB_2572981
Rabbit anti-DBP	Sigma-Aldrich	Sigma-Aldrich Cat# HPA019855; RRID:AB_1849545
Mouse anti-PCNA	Cell Signaling Technology	Cell Signaling Technology Cat# 2586; RRID:AB_2160343
Guinea pig anti-insulin	Agilent	Agilent Cat# A0564; RRID:AB_10013624
Mouse anti-glucagon	Abcam	Abcam Cat# ab10988; RRID:AB_297642
Goat anti-rabbit Alexa Fluor 488	Thermo Fisher Scientific	Thermo Fisher Scientific Cat# R37116; RRID:AB_2556544
Goat anti-guinea pig Alexa Fluor 488	Thermo Fisher Scientific	Thermo Fisher Scientific Cat# A-11073; RRID:AB_2534117
Goat anti-mouse Alexa Fluor 488	Thermo Fisher Scientific	Thermo Fisher Scientific Cat# A-11029; RRID:AB_138404
Goat anti-guinea pig Alexa Fluor 568	Thermo Fisher Scientific	Thermo Fisher Scientific Cat# A-11075; RRID:AB_2534119
Goat anti-mouse Alexa Fluor 555	Thermo Fisher Scientific	Thermo Fisher Scientific Cat# A-11075; RRID:AB_2534119
Goat anti-rabbit Alexa Fluor 633	Thermo Fisher Scientific	Thermo Fisher Scientific Cat# A-21052; RRID:AB_2535719
Goat anti-guinea pig Alexa Fluor 647	Thermo Fisher Scientific	Thermo Fisher Scientific Cat# A-21450; RRID:AB_2735091

**Chemicals, Peptides, and Recombinant Proteins**		

PowerUp SYBR Green Master Mix	Thermo Fisher Scientific	Thermo Fisher Scientific Cat# A25742
TaqMan Fast Advanced Master Mix	Thermo Fisher Scientific	Thermo Fisher Scientific Cat# 4444556
SERVA NB8; amsbio Cat# 17456.01	amsbio	amsbio Cat# 17456.01
VECTASHIELD HardSet with DAPI	Vector Laboratories	Vector Laboratories Cat# H-1500
Fura2-AM	Hello Bio	HelloBio HB0780-1mg
Phalloidin-488	Abcam	Abcam Cat# ab176753
Latrunculin B	Abcam	Abcam Cat# ab144291
DNaseI-594	Invitrogen	Invitrogen Cat# D12372

**Critical Commercial Assays**		

Ultrasensitive glucagon HTRF assay	Cisbio	Cisbio Cat# 62CGLPEG
Ultrasensitive insulin HTRF assay	Cisbio	Cisbio Cat# 62IN2PEG
Glucagon ELISA - 10 μL	Mercodia	Mercodia Cat#10-1281-01

**Experimental Models: Organisms/Strains**		

DBP^−/−^ mice	Prof Nancy Cooke, University of Pennsylvania	(Safadi et al., 1999)
Human pancreas sections	Exeter Archival Diabetes Biobank and Alberta Diabetes Institute IsletCore	https://foulis.vub.ac.be/
		https://www.epicore.ualberta.ca/isletcore/Default
		https://iidp.coh.org

**Software and algorithms**		

Prism 7	GraphPad software	N/A
MATLAB	Mathworks	N/A
R-Studio	R-Project	N/A
Igor Pro	WaveMetrics	N/A
Patchmaster	HEKA Electronics	N/A

**Other**		

Contour XT glucometer	N/A	Bayer
Zeiss LSM780 meta-confocal	N/A	Carl Zeiss Microscopy
Zeiss LSM880 meta-confocal	N/A	Carl Zeiss Microscopy
Crest spinning disk	N/A	Cairn Research
Nikon N-SIM S	N/A	Nikon Instruments
Applied Biosystems 7500/7900HT Real-Time PCR System	N/A	Applied Biosystems

### Resource Availability

#### Lead contact

Further information and requests for resources and reagents should be directed to and will be fulfilled by the Lead Contact, David J. Hodson (d.hodson@bham.ac.uk).

#### Materials availability

This study did not generate new unique reagents.

#### Data and code availability

This study did not generate any unique datasets or code.

### Experimental Model and Subject Details

#### Mouse models

DBP^−/−^ mice were generated using a PGK-promoter/neomycin cassette to disrupt exon 5 of the mouse *Gc* gene, as described (Safadi et al., 1999). We used these animals rather than the KOMP repository strain (Gc^tm1.1(KOMP)Vlcg^; MGI:5577272), since they have been subjected to thorough phenotypic validation and show loss of serum DBP protein, as well as 25(OH)[^3^H]D_3_ binding (Safadi et al., 1999). DBP^−/−^ mice were backcrossed for 10 generations onto a C57BL/6J background at the University California Los Angeles, before re-derivation of embryos into the University of Birmingham facility using C57BL/6J recipients. The line was refreshed every few months by backcrossing with non-sibling C57BL/6J purchased from Charles River UK. Animals were group-housed in a specific-pathogen free facility with ad lib access to regular chow (which contains 1000 U/kg cholecalciferol) and water. All studies were performed with 6-15 week-old male and female animals, and regulated by the Animals (Scientific Procedures) Act 1986 of the UK. Littermates were allocated to treatment groups in a randomized manner to ensure that all states were represented in the different experiment arms. Investigators were blinded to animal identity. Approval was granted by the University of Birmingham’s Animal Welfare and Ethical Review Body.

#### Human donors

Formalin-fixed paraffin-embedded pancreas sections were obtained from the Exeter Archival Diabetes Biobank (EADB) (https://foulis.vub.ac.be/) or the Alberta Diabetes Institute IsletCore (quality control and phenotyping data is available for each preparation via https://www.epicore.ualberta.ca/isletcore/Default and https://iidp.coh.org). All EADB samples were used with ethical permission from the West of Scotland Research Ethics Committee (ref: 15/WS/0258). Procurement of human pancreases was approved by the Human Research Ethics Board (Pro00013094; Pro00001754) at the University of Alberta and all families of organ donors provided written informed consent. Studies with human tissue were approved by the University of Birmingham Ethics Committee, as well as the National Research Ethics Committee (REC reference 16/NE/0107, Newcastle and North Tyneside, UK). Donor age, sex and BMI are reported in Table S1.

### Method Details

#### Glucose, insulin and pyruvate tolerance testing

Mice were fasted for 4-5 hr before intraperitoneal injection of either 2g/kg sterile-filtered D-glucose, 0.75 U/kg insulin or 2g/kg pyruvate, and tail vein bleed at 0, 15, 30, 60, 90 and 120 mins. Glucose levels were measured using a Contour XT glucometer (Bayer). For glucagon measures, animals were fasted for 4 hr, insulin injected at 0 min, and blood collected at 30 min. Non-responsive animals were excluded from analysis for both genotypes. Serum glucagon was assayed using a glucagon enzyme-linked immunosorbent assay (ELISA) kit (Mercodia Cat# 10-1281-01) (10 μl serum/sample used).

#### Vitamin D measures

Animals were bled under terminal anesthesia, before measurement of circulating concentrations of 25(OH)D and hormonal 1,25(OH)_2_D using liquid chromatography-tandem mass spectrometry methods, as described previously (Tamblyn et al., 2017).

#### Islet isolation and culture

Animals were euthanized by cervical dislocation, before isolation of islets using collagenase digestion (1 mg/ml, SERVA NB8; amsbio Cat# 17456.01) and Histopaque or Ficoll-Paque gradient separation. Islets were maintained at 37°C and 5% CO_2_ in RPMI medium containing 10% FCS, 100 units/mL penicillin, and 100 μg/mL streptomycin.

#### Gene expression

Relative mRNA abundance was determined using an Applied Biosystems 7500 or 7900HT instrument and PowerUp SYBR Green Master Mix (Thermo Fisher Scientific Cat# A25742) or TaqMan Fast Advanced Master Mix (Thermo Fisher Scientific Cat# 4444556). Fold-change mRNA expression was calculated versus *Ppia* or *Gusb* by using the 2^–ΔΔCt^ method. For primer sequences, see Key Resources Table.

#### Glucagon and insulin assays

Batches of 10 islets were pre-incubated in either 10 mM or 3 mM glucose for 1 hour at 37°C in buffer containing (in mmol/L) 120 NaCl, 4.8 KCl, 24 NaHCO_3_, 0.5 Na_2_HPO_4_, 5 HEPES, 2.5 CaCl_2_, 1.2 MgCl_2_ + 0.1% BSA. For glucagon secretion, islets were incubated in 10 mM, 0.5 mM or 0.5 mM glucose + 5 μM epinephrine for 1 hour at 37°C. Insulin was measured similarly, but using batches of 10 islets sequentially incubated in 3 mM glucose, 17 mM glucose and 17 mM glucose + 10 mM KCl for 30 minutes at 37°C. Total glucagon and insulin were extracted from islets lysed in acid ethanol. Glucagon and insulin concentrations were measured using specific ultrasensitive HTRF assay (glucagon; Cisbio Cat# 62CGLPEG) (insulin; Cisbio Cat# 62IN2PEG). In all cases, values are normalized against total glucagon/insulin for each individual experiment to account for differences in α-cell/β-cell proportion with treatment and islet size (Henquin, 2019).

#### Immunostaining of mouse tissue

Pancreata were fixed in 10% formalin overnight, before dehydration and wax embedding. Sections were blocked with PBS-T + 1% BSA for 1 hour and incubated with primary antibodies overnight at 4°C. Following washing in PBS-T + 0.1% BSA, secondary antibodies were applied for 2 hours at room temperature. Primary antibodies were rabbit anti-insulin 1:500 (Cell Signaling Technology Cat# 3014, RRID:AB_2126503), guinea pig anti-insulin 1:50 (Abcam Cat# ab7842, RRID:AB_306130), mouse monoclonal anti-glucagon 1:2000 (Sigma-Aldrich Cat# G2654, RRID:AB_259852), rabbit anti-glucagon 1:100 (Sigma-Aldrich Cat# SAB4501137, RRID:AB_10761583), mouse anti-somatostatin 1:1000 (Thermo Fisher Scientific Cat#14-9751-80, RRID:AB_2572981), rabbit anti-DBP 1:1000 (Sigma-Aldrich Cat# HPA019855, RRID:AB_1849545), guinea pig anti-PDX1 1:200 (Abcam Cat# ab47308, RRID:AB_777178), rabbit anti-MafA 1:1000 (Bethyl laboratories Cat# IHC-00352, RRID:AB_1279486), and mouse anti-PCNA 1:500 (Cell Signaling Technology Cat# 2586, RRID:AB_2160343). We note that the rabbit anti-DBP antibody (Sigma-Aldrich Cat# HPA019855, RRID:AB_1849545) was developed and validated by the Human Protein Atlas project, passing multiple quality controls (https://www.proteinatlas.org/ENSG00000145321-GC/antibody#protein_array). Specificity was further confirmed here using DBP^−/−^ tissue in which antibody staining was absent using non-fluorescent immunohistochemistry.

Secondary antibodies were goat anti-rabbit Alexa Fluor 633 (Thermo Fisher Scientific Cat# A-21052, RRID:AB_2535719), goat anti-rabbit Alexa Fluor 488 (Thermo Fisher Scientific Cat# R37116, RRID:AB_2556544), goat anti-guinea pig Alexa Fluor 488 (Thermo Fisher Scientific Cat# A-11073, RRID:AB_2534117), goat anti-mouse Alexa Fluor 488 (Thermo Fisher Scientific Cat# A-11029, RRID:AB_138404), goat anti-guinea pig Alexa Fluor 568 (Thermo Fisher Scientific Cat# A-11075, RRID:AB_2534119), all at 1:1000. Fixed islets were incubated with Phalloidin-488 (Abcam Cat# ab176753) and DNaseI-594 (Invitrogen Cat# D12372) for 2 hours at room temperature to stain F-actin and G-actin.

Images were captured using either Zeiss LSM780 or LSM880 meta-confocal microscopes, the latter equipped with an Airyscan super-resolution module. Excitation was delivered at λ = 488 nm, λ = 568 and λ = 633 nm for Alexa Fluor 488, Alexa Fluor 568 and Alexa Fluor 633, respectively. Emitted signals were detected using a GaAsP PMT detector at λ = 498–559, nm λ = 568–629 and λ = 633–735 nm for Alexa Fluor 488, Alexa Fluor 568 and Alexa Fluor 633, respectively. Super-resolution images were subjected to online deconvolution processing using Zen Black (Zeiss Microscopy).

#### Immunostaining of human tissue

Tissue was obtained from individuals with T1D and their age-matched controls. Donor details are provided in Table S1. Samples were dewaxed and rehydrated before antigen retrieval and blocking with 5% normal goat serum. Primary antibodies were guinea pig anti-insulin 1:700 (Agilent Cat# A0564, RRID:AB_10013624), mouse anti-glucagon 1:2000 (Abcam Cat# ab10988, RRID:AB_297642) or mouse monoclonal anti-glucagon 1:2000 (Sigma-Aldrich Cat# G2654, RRID:AB_259852), and rabbit anti-DBP 1:500 (Sigma-Aldrich Cat# HPA019855, RRID:AB_1849545). Secondary antibodies were goat anti-guinea pig Alexa Fluor 647 (Thermo Fisher Scientific Cat# A-21450, RRID:AB_2735091), goat anti-mouse Alexa Fluor 555 (Thermo Fisher Scientific Cat# A-11075, RRID:AB_2534119), and goat anti-rabbit Alexa Fluor 488 at 1:400 (Thermo Fisher Scientific Cat# R37116, RRID:AB_2556544).

Images were captured using Zeiss LSM780 and LSM880 meta-confocal microscopes, as above. Excitation was delivered at λ = 488 nm, λ = 568 nm and λ = 633 nm for Alexa Fluor 488, Alexa Fluor 555 and Alexa Fluor 647 nm, respectively. Emitted signals were detected using a GaAsP PMT detector at λ = 498–561 nm, λ = 564–617 nm and λ = 641–691 nm for Alexa Fluor 488, Alexa Fluor 555 and Alexa Fluor 647 nm, respectively.

Structured Illumination Microscopy (SIM) was performed using a Nikon N-SIM S microscope, equipped with an SR HP Apo TIRF 100x 1.49 NA/oil immersion objective and ORCA-Flash 4.0 sCMOS camera. Excitation was delivered at λ = 488 nm and λ = 568 nm for Alexa Fluor 488 and Alexa Fluor 555 nm, respectively. Emitted signals were detected at λ = 500-550 nm and λ = 570-640 nm for Alexa Fluor 488 and Alexa Fluor 555, respectively.

#### Analysis of α-cell and β-cell mass

Pancreatic sections for determination of α-cell and β-cell mass were stained as above, before scanning and digitization using a Zeiss Axio Scan.Z1. Excitation was delivered at λ = 453-485 nm and λ = 590-650 nm for Alexa Fluor 488 and Alexa Fluor 647, respectively. Emitted signals were detected using an Orca Flash 4.0 at λ = 507-546 nm and λ = 663-738 nm for Alexa Fluor 488 and Alexa Fluor 647, respectively. Overall, 408 separate images were captured for each pancreas section using a 20 x / 0.8 NA objective, before compilation into a single image using Zen lite 2012.

#### Ca^2+^ imaging

Islets were loaded with Fura2 (HelloBio HB0780-1mg) before imaging using a Crest X-Light spinning disk system coupled to a Nikon Ti-E base and 10 x / 0.4 air objective. Excitation was delivered at λ = 340 nm and λ = 385 nm using a FuraLED system, with emitted signals detected at λ = 470–550 nm. Traces were presented as the emission ratio at 340 nm and 385 nm (i.e., 340/385). HEPES-bicarbonate buffer was used, containing (in mmol/L) 120 NaCl, 4.8 KCl, 24 NaHCO_3_, 0.5 Na_2_HPO_4_, 5 HEPES, 2.5 CaCl_2_, 1.2 MgCl_2_, and 0.5–17 D-glucose.

#### Electrophysiology

Whole-cell currents were recorded in intact islets using the standard whole-cell configuration, as previously described (Briant et al., 2018). Measurements were performed using an EPC-10 patch-clamp amplifier and Patchmaster software (HEKA Electronics). Currents were filtered at 2.9 kHz and digitized at more than 10 kHz. Currents were compensated for capacitive transients and leak current subtraction was conducted. The extracellular solution consisted of (in mmol/L) 138 NaCl, 5.6 KCl, 1.2 MgCl_2_, 5 HEPES (pH 7.4 with NaOH), 2.6 CaCl_2_ and 1 D-glucose. The intracellular solution contained (in mmol/L) 125 KCl, 1 CaCl_2_, 1 MgCl_2_, 5 HEPES, 3 MgATP and 10 EGTA (KOH buffered). Recordings with an access resistance of < 50 mΩ were used for analysis in MATLAB. The logistic regression model identifying cell type was implemented in MATLAB, as previously described (Briant et al., 2017).

### Quantification and Statistical Analysis

#### Image analysis

F-actin, G-actin, glucagon and DBP expression levels were analyzed using integrated density (area x mean fluorescence intensity), which accounts for the influence of cell size on fluorophore emission intensity for a given pixel (i.e., intensity of *n* fluorescent molecules will increase as a function of area^-1^). Corrected total cell fluorescence (CTCF) was then calculated as follows: integrated density – (area of selected cell x mean background fluorescence) (Gavet and Pines, 2010). Quantification of α-cell, β-cell and δ-cell area and number was performed on binarized images using ImageJ (NIH) and Threshold, Nucleus Counter and Particle Analysis plugins.

Glucagon granule distribution was analyzed using the G-function, which measures the distance from any position to the nearest object of interest compared to a random distribution of the same measured objects (FIJI Spatial Statistic 2D/3D plugin) (Andrey et al., 2010). A left shift away from the mean ± 95% confidence intervals indicates a less random or more clustered organization.

Linear adjustments to brightness and contrast were applied to representative images, with intensity values maintained between samples to allow accurate cross-comparison. For super-resolution images, the following FIJI look-up-tables were used: NanoJ-Orange, cyan and magenta.

#### Statistical analysis

Statistical details of experiments can be found in the figure legends, No data were excluded unless the cells displayed a clear non-physiological state (i.e., impaired viability), and all individual data points are reported in the figures. The measurement unit (n number) is animal, batch of islets or donor, with experiments replicated independently at least three times.

Data normality was assessed using D’Agostino-Pearson test. Unpaired/paired Students t test or Mann-Whitney test were used for pairwise comparisons (two-sided). Multiple interactions were determined using one-way or two-way ANOVA followed by Tukey’s, Dunnett’s, Bonferonni’s or Sidak’s post hoc tests (accounting for degrees of freedom). Analyses were conducted using GraphPad Prism or Microsoft Excel software. Data are presented as mean ± SEM or SD, with individual datapoints shown where practicable.

## Supplemental Information

Document S1. Figures S1–S4 and Table S1

Document S2. Article plus Supplemental Information
