# Supplementary material for: Vitamin-D-Binding Protein Contributes to the Maintenance of α Cell Function and Glucagon Secretion
Source: Cell Rep. 2020 Jun 16;31(11):107761. doi: 10.1016/j.celrep.2020.107761 (PMC7302426; doi:10.1016/j.celrep.2020.107761)
Supplement: Document S1. Figures S1–S4 and Table S1 [file mmc1.pdf]

Cell Reports, Volume *31*

Supplemental Information

Vitamin-D-Binding Protein Contributes to the Maintenance of α Cell Function and Glucagon Secretion

Katrina Viloria, Daniela Nasteska, Linford J.B. Briant, Silke Heising, Dean P. Larner, Nicholas H.F. Fine, Fiona B. Ashford, Gabriela da Silva Xavier, Maria Jiménez Ramos, Annie Hasib, Federica Cuozzo, Jocelyn E. Manning Fox, Patrick E. MacDonald, Ildem Akerman, Gareth G. Lavery, Christine Flaxman, Noel G. Morgan, Sarah J. Richardson, Martin Hewison, and David J. Hodson

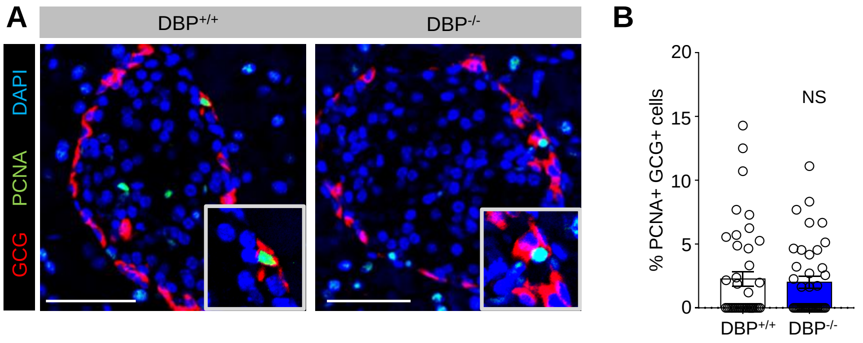

Figure S1, related to Figure 2: No changes in α-cell turnover are detected following loss of DBP

(A) Representative images showing PCNA and glucagon (GCG) staining in islets of $DBP^{+/+}$ and $DBP^{-/-}$ mice (n = 4 animals) (scale bar = 53 µm).

(B) The number of PCNA-positive α-cells is similar in islets of $DBP^{+/+}$ and $DBP^{-/-}$ mice (n = 4 animals).

Bar graph shows scatter plot with mean ± SEM. NS, non-significant. DBP, Vitamin D-binding protein; GCG, glucagon; PCNA, proliferating cell nuclear antigen.

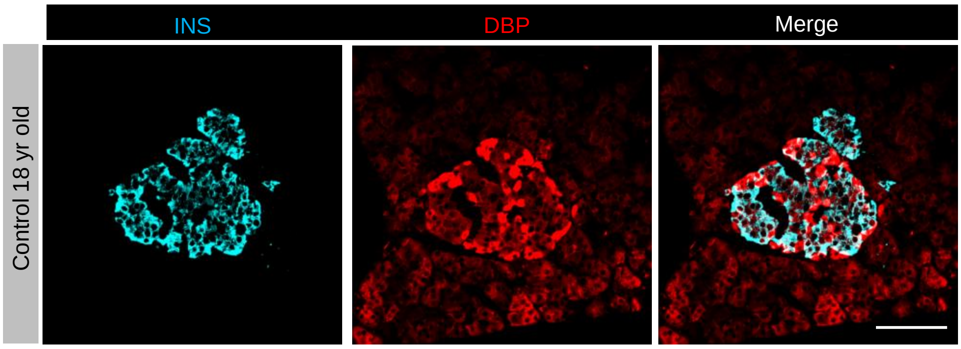

Figure S2, related to Figure 5: DBP is present at low levels in β-cells

DBP can be detected in insulin-positive cells, as well as in exocrine tissue, in control human donors. Due to the strength of DBP expression in α-cells, the image has been overexposed to allow visualization of DBP in the glucagon-negative compartment (scale bar = 42.5 µm) (representative images from n = 3 control donors). DBP, Vitamin D-binding protein; INS, insulin.

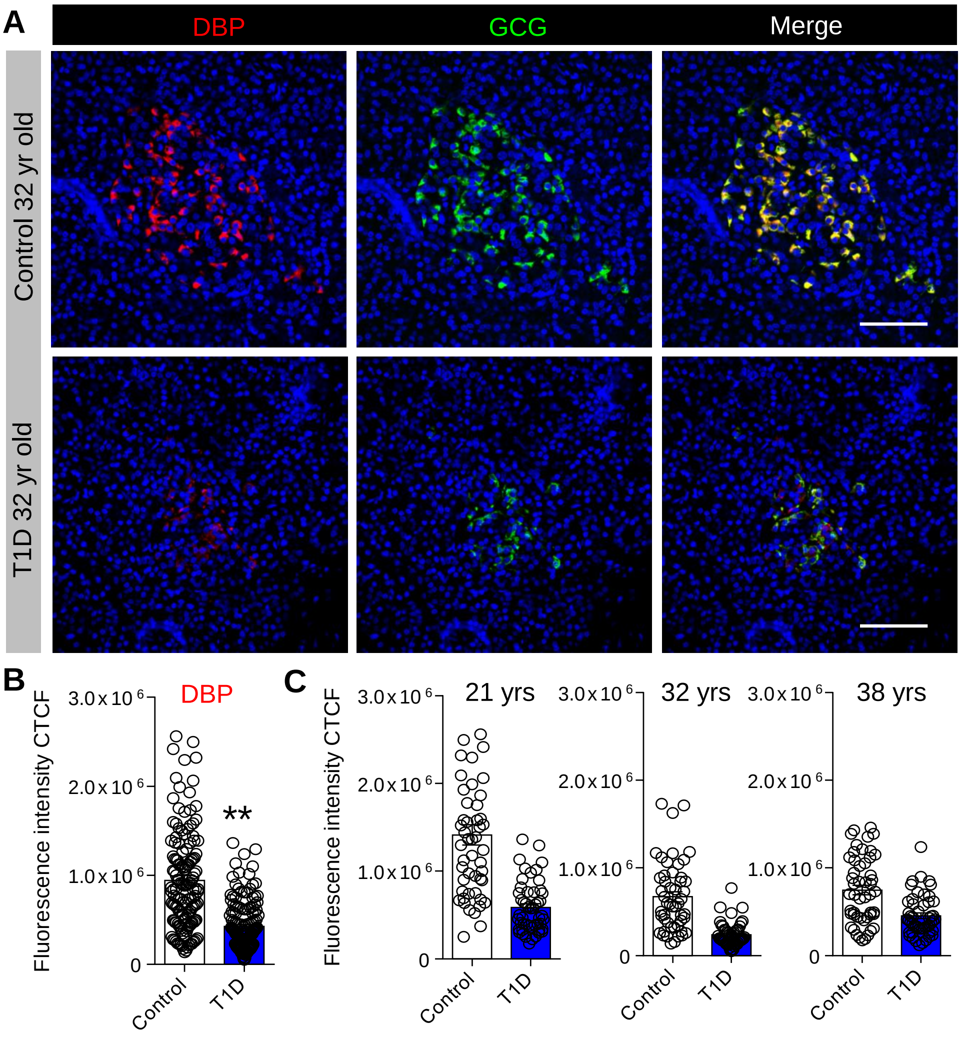

Figure S3, related to Figure 5: DBP is decreases in islets of donors with morelongstanding T1D.

(A) Representative images showing DBP expression in islets of donors with more longstanding T1D, together with their age-matched controls, both obtained from the IsletCore (Alberta) biobank (3 T1D donors and 3 age-matched controls).

(B and C) DBP levels are decreased in adult donors with longstanding T1D, as shown by mean (B) and individual donor (C) data (n = 150 cells, 30 islets, 3 T1D donors and 3 age-matched controls) (unpaired t-test). Scale bar = 42.5 µm.

Bar graphs show scatter plot with mean ± SEM. **P<0.01 and NS, non-significant. DBP, Vitamin D-binding protein; GCG, glucagon.

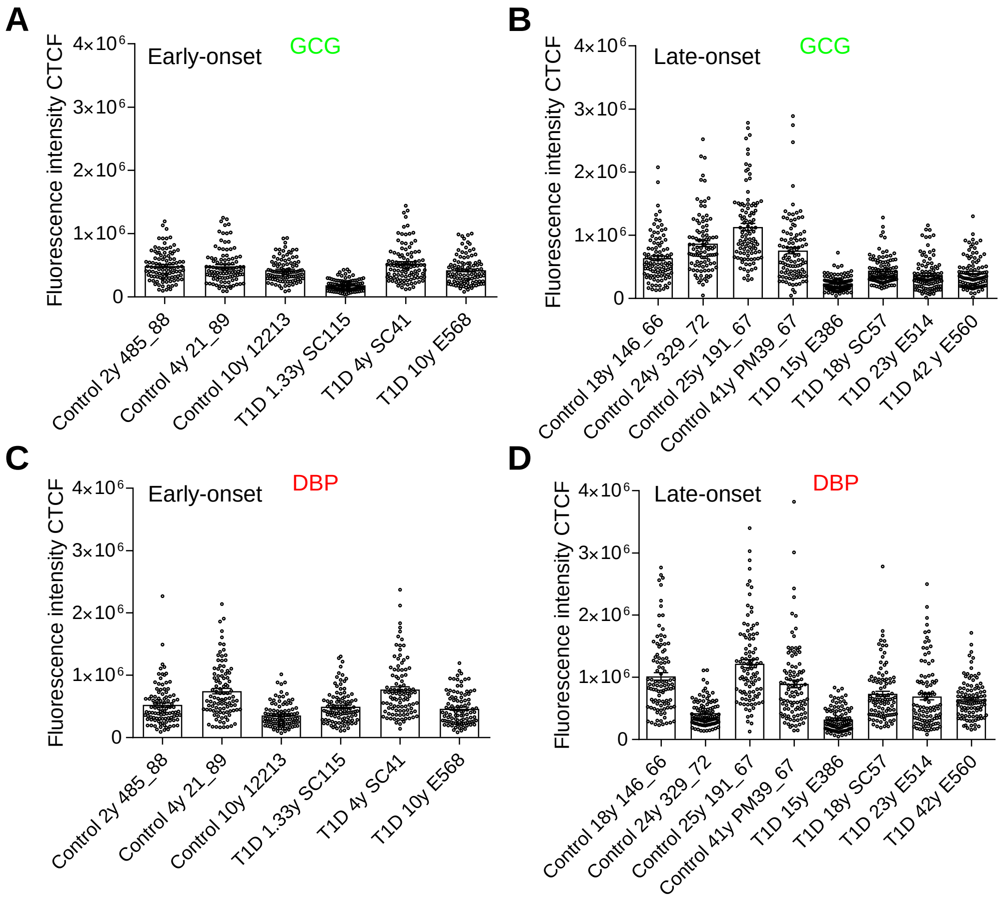

Figure S4, related to Figure 5: Individual donor glucagon and DBP expression levels

(A and B) Expression levels of glucagon in individual early-onset (≤ 10 yrs old) (A) and late-onset (≥ 15 yrs old) (B) T1D donors together with their age-matched controls (n = 100 cells, 10 islets from each donor).

(C and D) Expression levels of DBP in individual early-onset (C) and late-onset (D) T1D donors together with their age-matched controls (n = 100 cells, 10 islets from each donor).

Bar graphs show scatter plot with mean ± SEM. DBP, Vitamin D-binding protein; GCG, glucagon.

Tissue	Donor case ID	Age (years)	Sex	Cohort	Duration
Control	485/88	2	F	Exeter	
Control	21/89	4	F	Exeter	
Control	12213	10	unknown	Exeter	
Control	146/66	18	unknown	Exeter	
Control	329/72	24	unknown	Exeter	
Control	191_67	25	M	Exeter	
Control	447/71	32	F	Exeter	
Control	PM39/67	41	unknown	Exeter	
Control	330_71	47	M	Exeter	
Control	3730A/88	53	F	Exeter	
Control	9310/08	58	F	Exeter	
Control	224/66	67	M	Exeter	
Control	36/66	70	F	Exeter	
Control	424/67	70	M	Exeter	
Control	R187	21	M	Alberta	
Control	R294	32	M	Alberta	
Control	R044	38	M	Alberta	
T1D	SC115	1.33	F	Exeter	3 days
T1D	SC41	4	F	Exeter	3 wks
T1D	E568	10	M	Exeter	2 days
T1D	E386	15	M	Exeter	6 mths
T1D	SC57	18	F	Exeter	< 1 wk
T1D	E514	23	M	Exeter	2 wks
T1D	E560	42	n/a	Exeter	n/a
T1D	R015	21	M	Alberta	n/a
T1D	R079	32	M	Alberta	17 yrs
T1D	R035	38	F	Alberta	15 yrs

Table S1, related to Figure 5 and Figure 6: List of control and T1D donors used for immunohistochemical analyses in Figure 5 & 6. M, male; F, female; n/a, not available.
